# Supplementary material for: DNA topoisomerase IIIβ promotes cyst generation by inducing cyst wall protein gene expression in Giardia lamblia
Source: Open Biol. 2020 Feb 5;10(2):190228. doi: 10.1098/rsob.190228 (PMC7058931; doi:10.1098/rsob.190228)
Supplement: Table S1 to Fig S10 change.pdf [file rsob190228supp1.pdf]

Table S1. Genes up or down regulated by Top3β overexpression.

Number	Annotation	Orf number	Fold change (pPTop3β/5'ΔN5-Pac)*
1	VSP	113304	64.34
2	VSP	113163	39.80
3	Hypothetical protein	31366	14.44
4	Cyst wall protein 1	5638	11.94
5	Hypothetical protein	5206	9.48
6	Cyst wall protein 2	5435	9.11
7	Hypothetical protein	16622	8.65
8	Ceramide glucosyltransferase	11642	8.40
9	VSP	41472	8.33
10	Glucose 6-phosphate N-acetyltransferase	14259	7.98
11	VSP with INR	113450	7.86
12	High cysteine membrane protein Group 6	113512	7.50
13	VSP with INR	16501	7.50
14	VSP	114162	6.90
15	Hypothetical protein	27652	6.34
16	Hypothetical protein	14690	6.19
17	Hypothetical protein	3731	6.19
18	Hypothetical protein	4984	5.98
19	VSP, putative	114286	5.44
20	Hypothetical protein	11120	5.35
21	VSP	112867	5.17
22	Hypothetical protein	7374	5.08
23	Hypothetical protein	135270	5.07
24	Hypothetical protein	8505	4.93
25	Hypothetical protein	102575	4.45
26	VSP	11521	4.39
27	Hypothetical protein	9605	4.38
28	Hypothetical protein	115669	4.30
29	VSP presumed INR	137714	4.29
30	VSP with INR	11470	4.25
31	VSP	137708	3.89

32	Sugar transport family protein	9046	3.89
33	Hypothetical protein	32657	3.89
34	Hypothetical protein	28112	3.89
35	CEGP1 protein	17120	3.89
36	Adenylate cyclase	14367	3.58
37	Hypothetical protein	119703	3.45
38	Hypothetical protein	116201	3.44
39	Hypothetical protein	16078	3.37
40	Zinc finger domain	2116	3.35
41	Hypothetical protein	113303	3.32
42	VSP	115796	3.13
43	VSP, putative	118133	3.12
44	High cysteine membrane protein Group 1	32607	3.12
45	VSP	101765	3.11
46	Hypothetical protein	36883	3.03
47	Hypothetical protein	13878	2.98
48	Hypothetical protein	8960	2.96
49	Hypothetical protein	23934	2.96
50	Hypothetical protein	119224	2.92
51	Cathepsin B-like cysteine proteinase 3 precursor	114165	2.92
52	High cysteine membrane protein Group 6	114470	2.84
53	Protein 21.1	4846	2.73
54	Nicotinamide-nucleotide adenylyltransferase	92618	2.71
55	Hypothetical protein	35638	2.67
56	Hypothetical protein	101768	2.59
57	Hypothetical protein	11050	2.54
58	Hypothetical protein	2860	2.53
59	VSP	122566	2.51
60	Hypothetical protein	2605	2.49
61	VSP with INR	113439	2.48
62	VSP	112801	2.45
63	Glucosamine-6-phosphat	8245	2.44

	e deaminase		
64	Hypothetical protein	2692	2.41
65	Retinoic acid induced 17-like protein	11930	2.39
66	VSP with INR	115797	2.39
67	VSP	26894	2.37
68	Furin precursor putative serine protease	2897	2.37
69	UDP-glucose 4-epimerase	7982	2.37
70	High cysteine membrane protein Group 1	11309	2.36
71	Hypothetical protein	33672	2.33
72	Hypothetical protein	114210	2.31
73	Hypothetical protein	29757	2.29
74	Protein Kinase	4033	2.25
75	Protein 21.1	92983	2.21
76	Hypothetical protein	2404	2.21
77	Hypothetical protein	8325	2.21
78	Hypothetical protein	7598	2.17
79	Hypothetical protein	10425	2.14
80	Fatty acid elongase 1	92729	2.14
81	Hypothetical protein	11148	2.14
82	Hypothetical protein	117068	2.12
83	Hypothetical protein	14833	2.11
84	High cysteine protein	94003	2.10
85	DNA topoisomerase III	15190	2.10
86	Hypothetical protein	119599	2.09
87	Variant-specific surface protein	11690	2.08
88	Hypothetical protein	10552	2.06
89	Dolichol-phosphate mannosyltransferase, putative	3180	2.06
90	Variant-specific surface protein	9276	2.05
91	Hypothetical protein	15532	2.04

92	UDP-N-acetylglucosamine pyrophosphorylase	16217	2.03
93	Chorein	87358	2.01
94	DNA topoisomerase III	7615	0.02
95	VSP	137617	0.20
96	VSP	40571	0.24
97	VSP with INR	119707	0.24
98	VSP	34357	0.24
99	VSP	90215	0.24
100	VSP with INR	40592	0.32
101	VSP	41539	0.34
102	VSP	115047	0.35
103	Hypothetical protein	92625	0.37
104	VSP	114065	0.38
105	High cysteine membrane protein Group 3	112126	0.38
106	High cysteine protein	87706	0.39
107	VSP	111903	0.39
108	VSP	113093	0.39
109	VSP	99743	0.40
110	Protein 21.1	15965	0.40
111	VSP	121070	0.41
112	VSP	137612	0.42
113	VSP	116477	0.42
114	Cytosine deaminase, putative	2486	0.43
115	DNA-damage inducible protein DDI1-like	7718	0.43
116	VSP	34196	0.43
117	VSP	41401	0.44
118	Hypothetical protein	19870	0.44
119	VSP	112678	0.44
120	High cysteine membrane protein Group 3	114891	0.45
121	Hypothetical protein	125106	0.45
122	VSP	41349	0.45
123	VSP	118900	0.45

124	VSP	137611	0.46
125	Hypothetical protein	123980	0.46
126	Hypothetical protein	17241	0.47
127	High cysteine membrane protein Group 1	10659	0.47
128	VSP	137607	0.47
129	VSP	111874	0.48
130	VSP	101410	0.49
131	Kinase, NEK	9870	0.49
132	Hypothetical protein	31420	0.49
133	Hypothetical protein	99726	0.50

**p* values were determined to be <0.05 for groups in which the average means changed by a factor of ≥ 2.0 or ≤ 0.5.

Table S2. Oligonucleotides used for construction of plasmids and PCR.

Name	Sequence (5'--->3')
top3βF (PCR1F)	CACCATGATCCTTCTCATTGCA
top3βR (PCR1R)	TCTGTGTTTGCGACCCCT
top3βHAF	CAGACAGTATCTGAGTAC
HAR	AGCGTAATCTGGAACATCGTATGGGTA
cwp1F	ATGATGCTCGCTCTCCTT
cwp1R	TCAAGGCGGGGTGAGGCA
cwp2F	ATGATCGCAGCCCTTGTTCTA
cwp2R	CCTTCTGCGGACAATAGGCTT
cwp3F	ATGTTTTCTCTGCTTCTTCT
cwp3R	TCTGTAGTAGGGCGGCTGTA
myb2F	ATGTTACCGGTACCTTCTCAGC
myb2R	GGGTAGCTTCTCACGGGGAAG
ranF	ATGTCTGACCCAATCAGC
ranR	TCAATCATCGTCGGGAAG
top3βrealF	GGAGGGGGACAGACCAAG
top3βrealR	CGTGAGGGATTAGCCCAGT
cwp1realF	AACGCTCTCACAGGCTCCAT
cwp1realR	AGGTGGAGCTCCTTGAGAAATTG
cwp2realF	TAGGCTGCTTCCCACTTTTGAG
cwp2realR	CGGGCCCGCAAGGT
cwp3realF	GCAAATTGGATGCCAAACAA
cwp3realR	GACTCCGATCCAGTCGCAGTA
myb2realF	TCCCTAATGACGCCAAACG
myb2realR	AGCACGCAGAGGCCAAGT
ranrealF	TCGTCCTCGTCGGAAACAA
ranrealR	AACTGTCTGGGTGCGGATCT
18SrealF	AAGACCGCCTCTGTCAATCAA
18SrealR	GTTTACGGCCGGGAATACG
top3βNF	GGCCGGCTAGCTCGGTGTCGATGTGCCAAAGC

top3βMR	GGCCGACGCGTTCGGTGTCGATGTGCCAAAGC
top3β865F	CGCCCCTTGCCAATGA
top3β926R	GAGAGACCGAGCACCGTTGA
top3βm1F	CTGGATACATTTCTTTCCCAAGAACTGAGTC
top3βm1R	GACTCAGTTCTTGGGAAAGAAATGTATCCAG
top3βm2MR	GGCCGACGCGTAATAGAGGTAAACTTCGCGT
top3βm3MR	GGCCGACGCGTTGTTGCCAGGAAGTGCTTAG
top3β5HF	GGCGGAAGCTTTACTAACTATGACTCTAGGGC
top3β5NR	GGCGGCCATGGGAATTATTTTTAGCATCCCAG
top3β3XF	GGCGGCTCGAGTGACCATCAAGTGCTTGCTATTAT
top3β3KR	GGCGGGGTACCCTGGCTATCCTTGTAGCATCC
top3β-guide	GAGAGCGGGTACCCTAGCTTATTGAAAAAGCGAGAGGCCATTGAGATGACTCGCCTGATTGCAATAGCAAACAGTGTCTATAGTCTAATTGTGGACAACAGAGGGCTTATTGCAACGTTGATGACCAAGTTCAACAAGGGCGTCGCCTCCTATGAGCAGGTCATTCGGAATTTCGACGACCGGTAGCGTCCCCAGAGTAAACCATTTTAAATTGAAATAGGCGGTTGGAAATAAAAGCGCGCC**GACCTTCATGTGTACTT*CTG***GTTTTAGAGCTAGAAATAGCAAGTTAAAATAAGGCTAGTCCGTTATCAACTTGAAAAAGTGGCACCGAGTCGGTGCTTTTTTGAATTCGAGAGCG, underlined region is U6 promoter, bold region is for annealing, underlined and bold region is upstream 3nt of PAM, other region is scaffold RNA
top3βm2R	AATAGAGGTAAACTTCGCGT
top3βm3R	TGTTGCCAGGAAGTGCTTAG
PCR2F	AAGGACCGCGCGACCTGG
PCR2R	CGTCTCCACATGGTATGACCG
top3β5F	TTGGGGGATGATGGTATCTTC
top3β5R	GCATCCCAGCCCTCAGCC
18S5F	CCAAAAAAGTGTGGTGCAGG
18S5R	GCCGGGCGCGGGCGCCGCGG
cwp15F	CAACGGCTTACTAAATCATTCTCTTG
cwp15R	TTCTGTGTTTCTTGATCTGAGAGTTGT
cwp25F	CACTTTGATGAGAGCATGGG
cwp25R	TTAGTTCATATCTTAAGTTA
cwp35F	TGGGGGAGATAGGAGAATAC

cwp35R	ATCAGTAGTAACTTATTTTTTGGGAAAGAC
myb25F	TGGCTATGTATTTTTTTCTTCTTCTACAGCT
myb25R	TAGCAGTACAGAGTAATTATTATTTTAGTA
U65F	TTGAGATGACTCGCCTGATTG
U65R	GAAATTCCGAATGACCTGCTC
iscsF	CACCATGATTTACCTGGACAAC
iscsR	GTCATGCTTCCACTCTAT
bipF	CACCATGACGTCTAGTCACGTTAA
bipR	GAGTTCATCTTTTTCTGCAT

Figure S1

```
Gl15190     1 ----------------------------------MILLIAEKPSIAEMISRNYGEG---A
Gl7615      1 -------------------------------MNRVILCVTEKNSVAAEVSNVLSKGSYSK
HsTOP3b     1 --------------------------------MKTVLMVAEKPSLAQSIAKILSRGSLSS
MmTOP3b     1 --------------------------------MKTVLMVAEKPSLAQSIAKILSRGNMSS
HsTOP3a     1 MIFPVARYALRWLRRPEDRAFSRAAMEMALRGVRKVLCVAEKNDAAKGIADLLSNGRMRR
MmTOP3a     1 MIFPVTLLAFQWHRRPGGRALSRAAMEVAFRGVRKVLCVAEKNDAAKGIADLLSNGRMRR
ScTOP3      1 ---------------------------------MKVLCVAEKNSIAKAVSQILGGGRSTS
EcTOP1      1 --------------------------------MGKALVIVESPAKAKTINKYLGSDYVVK
TmTOP1      1 -----------------------------MSKKVKKYIVVESPAKAKTIKSILGNEYEVF
EcTOP3      1 ----------------------------------MKLFIAEKPAVANDIVKALGGNFTRH

Gl15190    24 KKLDNVSFPTYTFVQSFE-----GSKETFMCTSVAGHVFEIDFDAELN-GSSVPQERLFE
Gl7615     30 KQLVQYFNEYKFTHSEEE----HGPAQQYVVVHAQGHMLELE-PDEGYEWGKCSPSDLFT
HsTOP3b    29 HKGLNGACSVHEYTGTFA-----GQPVRFKMTSVCGHVMTLDFLGKYNKWDKVDPAELFS
MmTOP3b    29 HKGLNGACSVHKYTGTFA-----GQPVHFKMTSVCGHVMTLDFLGKYNKWDKVDPAELFS
HsTOP3a    61 REGLSKFNKIYEFDYHLY-----GQNVTMVMTSVSGHLLAHDFQMQFRKWQSCNPLVLFE
MmTOP3a    61 KEGLSKFNKIYEFDYHLY-----GQNVTMIMTSVSGHLLAHDFQMQFRKWQSCNPLVLFE
ScTOP3     28 RDSGYMYVKNYDFMFSGFPFARNGANCEVTMTSVAGHLTGIDFSHDSHGWGKCAIQELFD
EcTOP1     29 SSVGHIRDLPTSGSAAKK--------SADSTSTKTAKKPKKDERGALVNRMGVDPWHNWE
TmTOP1     32 ASMGHIIDLPKS-------------------------------------KFGVDLEKDFE
EcTOP3     27 DGWFESDNTIVTNCFGHI---------------IESQPPENYNPEYKEWKIETLPLRLYP

Gl15190    78 RGHVHYSFTDSGSTVAKHLKSIGGKAMQLILCLDNDREGENICFEVLKVLKPTLRSDCR-
Gl7615     85 CGVHFKANAVF---KKHVLRPNAAETNVLVLMLDADREGENIGYDIIEIFCSVLPADTLL
HsTOP3b    84 QAPTEKKEANPKLNMVKFLQVEGRGCDYIVLWLDCDKEGENICFEVLDAVLPVMNKAHGG
MmTOP3b    84 QAPTEKKEANPKLNMVKFLQVEGRGCDYVVLWLDCDKEGENICFEVLDAVLPVMNNAHNG
HsTOP3a   116 AEIEKYCPENF-VDIKKTLERETRQCQALVIWTDCDREGENIGFEIIHVCK---AVKPNL
MmTOP3a   116 AEIEKYCPENF-IDIKKTLERETHHCQALVIWTDCDREGENIGFEIIHVCK---AVKPNL
ScTOP3     88 APLNEIMNNNQ-KKIASNIKREARNADYLMIWTDCDREGEYIGWEIWQEAKRGNRLIQND
EcTOP1     81 AHYEVLPGKEK---VVSELKQLAEKADHIYLATDLDREGEAIAWHLREVIGGDDARYSR-
TmTOP1     55 PEFAVIKGKEK---VVEKLKDLAKKGE-LLIASDMDREGEAIAWHIARVTN-TLGRKNR-
EcTOP3     72 VKYQPVESAEKQVKTIIELIR-RADVTEIIHAGDPDDEGQLLVDEVLEYAGNTKPVKRVL

Gl15190   137 --------------------------------------VRRARFSAVTKAEIQNAFRNLD
Gl7615    142 DIRPARLPKIRSPTEHTLRHILAADHVPSQTSVKRTIIVKRARFFGLTYPELTSAVYNAG
HsTOP3b   144 EK-----------------------------------TVFRARFSSITDTDICNAMACLG
MmTOP3b   144 EK-----------------------------------TVFRARFSSITDTDICNAMTRLS
HsTOP3a   172 Q-------------------------------------VLRARFSEITPHAVRTACENLT
MmTOP3a   172 R-------------------------------------VLRARFSEITPHAVRTACENLT
ScTOP3    147 Q-------------------------------------VYRAVFSHLERQHILNAARNPS
EcTOP1    137 -----------------------------------------VVFNEITKNAIRQAFNKPG
TmTOP1    109 -----------------------------------------IVFSEITPRVIREAVKNPR
EcTOP3    131 IN-------------------------------------------DNTLPAVKKALANPK

Gl15190   159 KPD--QNLSDAVEARQELDLKIGVAFTRFQTAHLHAQFA----------------DLQSS
Gl7615    202 PID--MNVVDAVDVRQFTDLRLGYSITRLQTDKLRSSFIDYRTG----------AFKAKK
HsTOP3b   169 EPD--HNEALSVDARQELDLRIGCAFTRFQTKYFQGKYG----------------DLDSS
MmTOP3b   169 EPD--HNEALSVDARQELDLRIGCAFTRFQTKYFQGKYG----------------DLDSS
HsTOP3a   195 EPD--QRVSDAVDVRQELDLRIGAAFTRFQTLRLQRIFP---------------EVLAEQ
MmTOP3a   195 EPD--QRVSDAVDVRQELDLRIGAAFTRFQTLRLQRIFP---------------EVLAEQ
ScTOP3    170 RLD--MKSVHAVGTRIEIDLRAGVTFTRLLTETLRNKLRNQATMTKDGAKHRGGNKNDSQ
EcTOP1    156 ELN--IDRVNAQQARRFMDRVVGYMVSPLLWKKIARGLS---------------------
TmTOP1    128 EID--MKKVRAQLARRILDRIVGYSLSPVLWRNFKSNLS---------------------
EcTOP3    148 NNRDFRGLYLKALARSVADAIYGLSMTRAYTIPAKTKGYKG-------------------

Gl15190   201 VISYGPCQTPTLSFCVS---------------------------RHDEIQRFRPKQIFSL
Gl7615    250 PISFGPCQVPTLGLVHSNDLLSLKNHQSSSFTLHLVCDPRPLQTHSGSVFKGLADALKRW
HsTOP3b   211 LISFGPCQTPTLGFCVE---------------------------RHDKIQSFKPETYWVL
MmTOP3b   211 LISFGPCQTPTLGFCVE---------------------------RHDKIQSFKPETYWVL
HsTOP3a   238 LISYGSCQFPTLGFVVE---------------------------RFKAIQAFVPEIFHRI
MmTOP3a   238 LISYGSCQFPTLGFVVE---------------------------RFKAIQAFVPEVFHKI
ScTOP3    228 VVSYGTCQFPTLGFVVD---------------------------RFERIRNFVPEEFWYI
EcTOP1    193 ---AGRVQSVAVRLVVE---------------------------REREIKAFVPEEFWEV
TmTOP1    165 ---AGRVQSATLKLVCD---------------------------REREILRFVPKKYHRI
EcTOP3    189 VLSVGRVQTPVLGLIVN---------------------------RTRANKNHKSSFYYTM
```

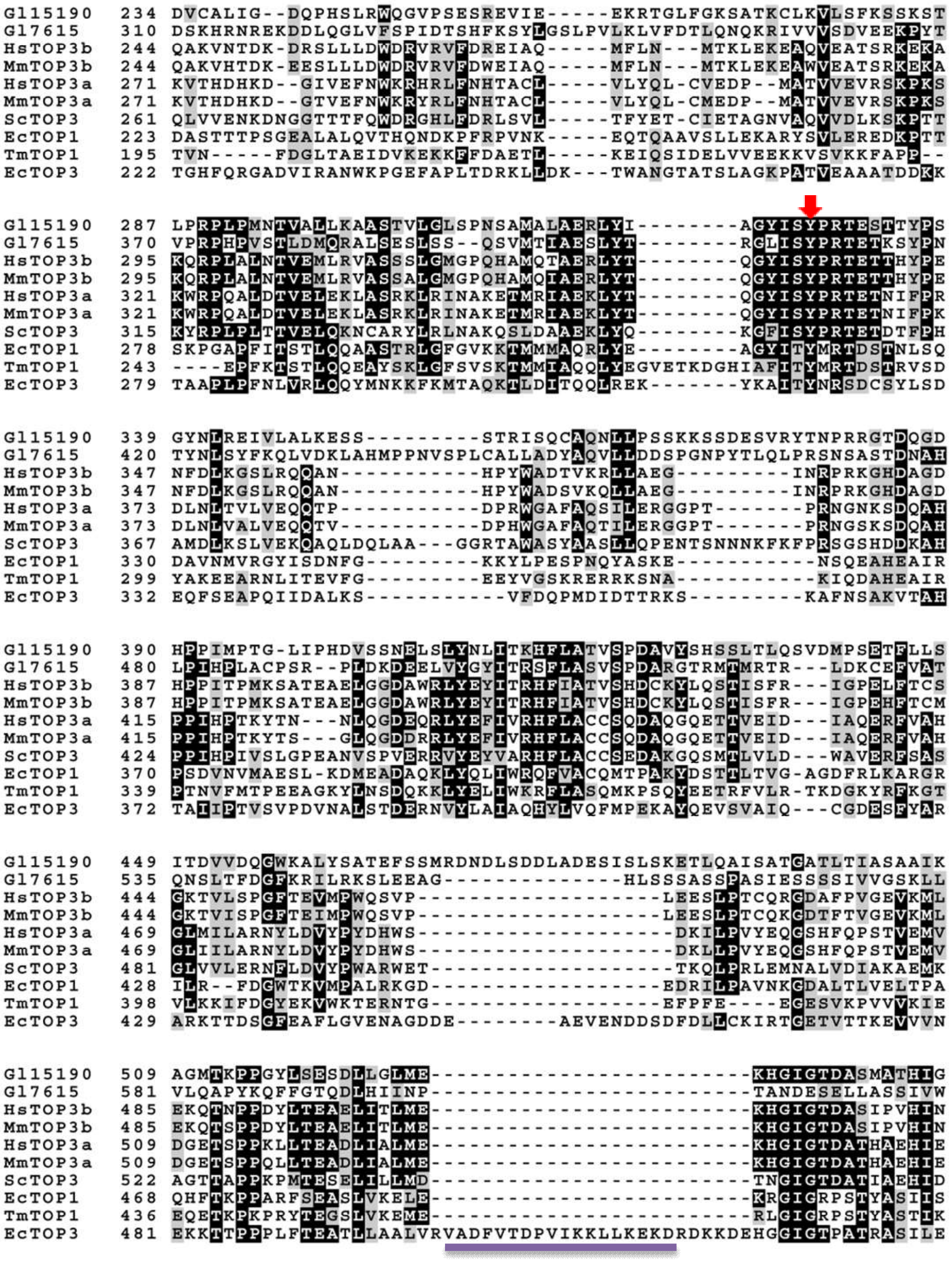

Gl15190 234 DVCALIG--DQPHSLRWQGVPSESREVIE-----EKRTGLFGKSATKCLKVLSFKSSKST
Gl7615 310 DSKHRNREKDDLQGLVFSPIDTSHFKSYLGSLPVLKLVFDTLQNQKRIVVVSDVEEKPYT
HsTOP3b 244 QAKVNTDK-DRSLLLDWDRVRVFDREIAQ-----MFLN---MTKLEKEAQVEATSRKEKA
MmTOP3b 244 QAKVHTDK-EESLLLDWDRVRVFDWEIAQ-----MFLN---MTKLEKEAWVEATSRKEKA
HsTOP3a 271 KVTHDHKD--GIVEFNWKRHRLFNHTACL-----VLYQL-CVEDP--MATVVEVRSKPKS
MmTOP3a 271 KVTHDHKD--GTVEFNWKRYRLFNHTACL-----VLYQL-CMEDP--MATVVEVRSKPKS
ScTOP3 261 QLVVENKDNGGTTTFQWDRGHLFDRLSVL-----TFYET-CIETAGNVAQVVDLKSKPTT
EcTOP1 223 DASTTTPSGEALALQVTHQNDKPFRPVNK-----EQTQAAVSLLEKARYSVLEREDKPTT
TmTOP1 195 TVN-----FDGLTAEIDVKEKKFFDAETL-----KEIQSIDELVVEEKKVSVKKFAPP--
EcTOP3 222 TGHFQRGADVIRANWKPGEFAPLTDRKLLDK---TWANGTATSLAGKPATVEAAATDDKK

Gl15190 287 LPRPLPMNTVALLKAASTVLGLSPNSAMALAERLYI--------AGYISYPRTESTTYPS
Gl7615 370 VPRPHPVSTLDMQRALSESLSS--QSVMTIAESLYT--------RGLISYPRTETKSYPN
HsTOP3b 295 KQRPLALNTVEMLRVASSSLGMGPQHAMQTAERLYT--------QGYISYPRTETTHYPE
MmTOP3b 295 KQRPLALNTVEMLRVASSALGMGPQHAMQIAERLYT--------QGYISYPRTETTHYPE
HsTOP3a 321 KWRPQALDTVELEKLASRKLRINAKETMRIAEKLYT--------QGYISYPRTETNIFPR
MmTOP3a 321 KWRPQALDTVELEKLASRKLRINAKETMRIAEKLYT--------QGYISYPRTETNIFPK
ScTOP3 315 KYRPLPLTTVELQKNCARYLRLNAKQSLDAAEKLYQ--------KGFISYPRTETDTFPH
EcTOP1 278 SKPGAPFITSTLQQAASTRLGFGVKKTMMMAQRLYE--------AGYITYMRTDSTNLSQ
TmTOP1 243 ----EPFKTSTLQQEAYSKLGFSVSKTMMIAQQLYEGVETKDGHIAFITYMRTDSTRVSD
EcTOP3 279 TAAPLPFNLVRLQQYMNKKFKMTAQKTLDITQQLREK-------YKAITYNRSDCSYLSD

Gl15190 339 GYNLREIVLALKESS---------STRISQCAQNLLPSSKKSSDESVRYTNPRRGTDQGD
Gl7615 420 TYNLSYFKQLVDKLAHMPPNVSPLCALLADYAQVLLDDSPGNPYTLQLPRSNSASTDNAH
HsTOP3b 347 NFDLKGSLRQQAN-----------HPYWADTVKRLLAEG---------INRPRKGHDAGD
MmTOP3b 347 NFDLKGSLRQQAN-----------HPYWADSVKQLLAEG---------INRPRKGHDAGD
HsTOP3a 373 DLNLTVLVEQQTP-----------DPRWGAFAQSILERGGPT-------PRNGNKSDQAH
MmTOP3a 373 DLNLVALVEQQTV-----------DPHWGAFAQTILERGGPT-------PRNGSKSDQAH
ScTOP3 367 AMDLKSLVEKQAQLDQLAA---GGRTAWASYAASLLQPENTSNNNKFKFPRSGSHDDKAH
EcTOP1 330 DAVNMVRGYISDNFG---------KKYLPESPNQYASKE----------NSQEAHEAIR
TmTOP1 299 YAKEEARNLITEVFG---------EEYVGSKRERRKSNA-----------KIQDAHEAIR
EcTOP3 332 EQFSEAPQIIDALKS-----------VFDQPMDIDTTRKS---------KAFNSAKVTAH

Gl15190 390 HPPIMPTG-LIPHDVSSNELSLYNLITKHFLATVSPDAVYSHSSLTLQSVDMPSETFLLS
Gl7615 480 LPIHPLACPSR--PLDKDEELVYGYITRSFLASVSPDARGTRMTMRTR---LDKCEFVAT
HsTOP3b 387 HPPITPMKSATEAELGGDAWRLYEYITRHFIATVSHDCKYLQSTISFR---IGPELFTCS
MmTOP3b 387 HPPITPMKSATEAELGGDAWRLYEYITRHFIATVSHDCKYLQSTISFR---IGPEHFTCM
HsTOP3a 415 PPIHPTKYTN---NLQGDEQRLYEFIVRHFLACCSQDAQGQETTVEID---IAQERFVAH
MmTOP3a 415 PPIHPTKYTS---GLQGDDRRLYEFIVRHFLACCSQDAQGQETTVEID---IAQERFVAH
ScTOP3 424 PPIHPIVSLGPEANVSPVERRVYEYVARHFLACCSEDAKGQSMTLVLD---WAVERFSAS
EcTOP1 370 PSDVNVMAESL-KDMEADAQKLYQLIWRQFVACQMTPAKYDSTTLTVG-AGDFRLKARGR
TmTOP1 339 PTNVFMTPEEAGKYLNSDQKKLYELIWKRFLASQMKPSQYEETRFVLR-TKDGKYRFKGT
EcTOP3 372 TAIIPTVSVPDVNALSTDERNVYLAIAQHYLVQFMPEKAYQEVSVAIQ---CGDESFYAR

Gl15190 449 ITDVVDQGWKALYSATEFSSMRDNDLSDDLADESISLSKETLQAISATGATLTIASAAIK
Gl7615 535 QNSLTFDGFKRILRKSLEEAG--------------HLSSSASSPASIESSSSIVVGSKLL
HsTOP3b 444 GKTVLSPGFTEVMPWQSVP-------------------LEESLPTCQRGDAFPVGEVKML
MmTOP3b 444 GKTVISPGFTEIMPWQSVP-------------------LEESLPTCQKGDTFTVGEVKML
HsTOP3a 469 GLMILARNYLDVYPYDHWS--------------------DKILPVYEQGSHFQPSTVEMV
MmTOP3a 469 GLIILARNYLDVYPYDHWS--------------------DKLLPVYEQGSHFQPSTVEMV
ScTOP3 481 GLVVLERNFLDVYPWARWET-------------------TKQLPRLEMNALVDIAKAEMK
EcTOP1 428 ILR--FDGWTKVMPALRKGD------------------EDRILPAVNKGDALTLVELTPA
TmTOP1 398 VLKKIFDGYEKVWKTERNTG------------------EFPFE----EGESVKPVVVKIE
EcTOP3 429 ARKTTDSGFEAFLGVENAGDDE--------AEVENDDSDFDLLCKIRTGETVTTKEVVVN

Gl15190 509 AGMTKPPGYLSESDLLGLME-------------------------KHGIGTDASMATHIG
Gl7615 581 VLQAPYKQFFGTQDLHIINP-------------------------TANDESELLASSIVW
HsTOP3b 485 EKQTNPPDYLTEAELITLME-------------------------KHGIGTDASIPVHIN
MmTOP3b 485 EKQTSPPDYLTEAELITLME-------------------------KHGIGTDASIPVHIN
HsTOP3a 509 DGETSPPKLLTEADLIALME-------------------------KHGIGTDATHAEHIE
MmTOP3a 509 DGETSPPQLLTEADLIALME-------------------------KHGIGTDATHAEHIE
ScTOP3 522 AGTTAPPKPMTESELILLMD-------------------------TNGIGTDATIAEHID
EcTOP1 468 QHFTKPPARFSEASLVKELE-------------------------KRGIGRPSTYASIIS
TmTOP1 436 EQETKPKPRYTEGSLVKEME-------------------------RLGIGRPSTYASTIK
EcTOP3 481 EKKTPPPLFTEATLLAALVRVADFVTDPVIKKLLKEKDRDKDEHGGIGTPATRASILE

```
Gl15190   544  NIVTRAYVELRVTGRRRCLVPTSMGISLIHGYQLIDGDLS--SPQLRASIERDVTRIAEG
Gl7615    616  DGDAQSLVIDIDSSDESEQIHAEENVHPPEPVMVNDREDP---SNSTSQFSTELVPLPMK
HsTOP3b   520  NICQRNYVTVESGRR---LKPTNLGIVLVHGYYKIDAELV--LPTIRSAVEKQLNLIAQG
MmTOP3b   520  NICQRNYVTVESGRR---LKPTNLGIVLVHGYYKIDAELV--LPTIRSAVEKQLNLIAQG
HsTOP3a   544  TIKARMYVG---LTPDKRFLPGHLGMGLVEGYDSMGYEMS--KPDLRAELEADLKLICDG
MmTOP3a   544  TIKARMYVG---LTSDKRFLPGHLGMGLVEGYDSMGYEMS--KPDLRAELEADLKLICEG
ScTOP3    557  KIQVRNYVRSEKVGKETYLQPTTLGVSLVHGFEAIGLEDSFAKPFQRREMEQDLKKICEG
EcTOP1    503  TIQDRGYVR----VENRRFYAEKMGEIVTDRLEENFRELMN--YDFTAQMENSLDQVANH
TmTOP1    471  LLLNRGYIK----KIRGYLYPTIVGSVVMDYLEKKYSDVVS--VSFTAEMEKDLDEVEQG
EcTOP3    541  TLKKRNYIT----LEKGKLIPTDTGYALIDALPDIAVNPD-----MTALWAEKQTLIENG

Gl15190   602  KIRKDVLVNQVLSKFLTKFLHFKQNIGKLE----------ALLNAKFTSIKKAGRPFILC
Gl7615    673  ESALLLAMDQHGIGTDATMSDHIALITDR-----------EYINSKKQVVSLGAELLQFY
HsTOP3b   575  KADYRQVLGHTLDVFKRKFHYFVDSIAGMD----------ELMEVSFSPLAATGKPLSRC
MmTOP3b   575  KADYHQVLGHTLDIFKRKFHYFVDSIAGMD----------ELMEVSFSPLAATGKPLSRC
HsTOP3a   599  KKDKFVVLRQQVQKYKQVFIEAVAKAKKLDEALAQYFGNGTELAQQEDIYPAMPEPIRKC
MmTOP3a   599  KKDKFQVLRQQVQKYKQVFIEAVAKAKKLDEALSQYLGERTEMAQQEEIYPAMPEPVRKC
ScTOP3    617  HASKTDVVKDIVEKYRKYWHKTNACKN-------------TLLQVYDRVKASM-------
EcTOP1    557  EAEWKAVLDHFFSDFTQQLDKAEKDPEEGGM-------RPNQMVLTSIDCPTCGRKMGIR
TmTOP1    525  KKTDKIVLREFYESFSSVFDRNDR---------------------IVVDFPTN-------
EcTOP3    592  EMTIEQFVDELYNDLIPMISNANS------------------AEIKVSPSAPSGQSQRLS

Gl15190   652  GECHRYTDLIEQYPPRVYCVTCDKLYTIPMRGTFIEIPSRKCPYDGWPLILHMAEATQKR
Gl7615    722  KQTPMGVQALSCSYRSMLEHGMHLICEGQIDCKSVHDDCIRWGMKLYDSIDRLQVIKVG-
HsTOP3b   625  GKCHRFMKYIQAKPSRLHCSHCDETYTLPQNGTIKLYKELRCPLDDFELVLWSSGSRGKS
MmTOP3b   625  GKCHRFMKYIQAKPSRLHCSHCDETYTLPQNGTIKLYKELRCPLDDFELVLWSSGSRGKS
HsTOP3a   659  PQCNKDMVLKTKKNGGFYLSCMGFPECRSAVWLPDSVLEASRDSSVCPVCQPHPVYRLKL
MmTOP3a   659  PQCNKDMVLKTKKSGGFYLSCMGFPECRSAVWFPDSVLEASRDNSVCSVCQPPPVYRLKL
ScTOP3         ------------------------------------------------------------
EcTOP1    610  TASTGVFLGCSGYALPPKERCKTTINLVPENEVLNVLEGEDAETNALRAKRRCPKCGTAM
TmTOP1    557  -----------------------------------------------------QKCS---
EcTOP3    634  SPCP--------------------------------------------------------

Gl15190   712  TFFCLHCYTFGLNKEEALTQLQTVAADGSTDIEQTKAITCGMCLNSLCQMSLLKTQVGAC
Gl7615    781  ----------------------------------------GYGGYRMLNEKPKKTTIKRK
HsTOP3b   685  YPLCPYCYNHPPFR------------------DMKKGMGCNECTHPSCQHSLSMLGIGQC
MmTOP3b   685  YPLCPYCYNHPPFR------------------DMKKGMGCNECTHPTCQHSLSMLGIGQC
HsTOP3a   719  KFKRGSLPPTMPLEFVCCIGGCDDTLREILDLRFSGGPPRASQPSGRLQANQSLNRMDNS
MmTOP3a   719  KFKRGSLPPAMPLEFVGCIGGCDETLKEIFGLRFPRALPRASQPSGHLQASQALNRMDSS
ScTOP3         ------------------------------------------------------------
EcTOP1    670  DSYLIDPKRKLHVCGNNPTCDGYEIEEGEFRIKGYDG-PIVECEKCGSEMHLKMGRFGKY
TmTOP1    561  ---------------------------------------------CGKEMRLSFGKYGFY
EcTOP3    638  --------------------------------------------SCGKQIVIRPK-----

Gl15190   772  WHCNADGRPMLSVNRDGPDSSPGATNDTLAVARSPEAQEQTPEKSAELASAPVQPKKISI
Gl7615    801  YHSATDGTALVSKRR--------------------------------QKEAQPVRVRGTF
HsTOP3b   727  VECESG----------------------------------------VLVLDPTSGPKWKV
MmTOP3b   727  VECENG----------------------------------------VLVLDPTSGPKWKV
HsTOP3a   779  QH--PQPADSRQTGSSKALAQTLPPPTAAGESNSVTCNCGQEAVLLTVRKEGPNRGRQFF
MmTOP3a   779  QHNLSQPLVNRHTRPSKTVAQALLPPTTAGESNSVTCNCGREAVLLTVRKQGPNQGRHFY
ScTOP3         ------------------------------------------------------------
EcTOP1    729  MACTNEECKNTRKILRNGEVAP------------------PKEDPVPLPELPCEKSDAYF
TmTOP1    576  LKC---ECGKTRSVK-NDEIA---------------------------------------
EcTOP3    649  ----------------------------------------------------------SY

Gl15190   832  TIKPQAKISLNIPQANKSSGSASAVPSPNK-------ASSLAELVGGYLCLDVGTARRLP
Gl7615    829  TCCGERCSRNSLAGSCAACGKAFQIPRTDG---------------VIVVLNEECDNCGLS
HsTOP3b   747  ACNKCNVVAHCFENAHRVRVSADTCSVCEA-------ALLDVDFNKAKSPLPGDETQHMG
MmTOP3b   747  ACNTCNVVAHCFENAHRVRVSADTCNTCEA-------ALLDVDFNKAKSPLPGNETQHTG
HsTOP3a   837  KCNGGSCNFFLWADSPNPGAGGPPALAYRPLGASLGCPPGPGIHLGGFGNPGDGSGSGTS
MmTOP3a   839  KCSNGDCNFFLWADSSHSTGGGTPTSASGPPGSSVGCPSSVGSHMDGFGSLGSDSDGGTP
ScTOP3         ------------------------------------------------------------
EcTOP1    771  VLRDGAAGVFLAANTFPKSRETRAPLVEELYRFRDRLPEKLRYLADAPQQDPEGNKTMVR
TmTOP1    593  VIDDGK--IFLGR----KDSESGSP--------------------DGRSVEGKGN-----
EcTOP3    651  SCTGCEFKIWNEFSGKKITQAQAEKLIKSG----------KTDLIKGFKKKSGGTYDAVL
```

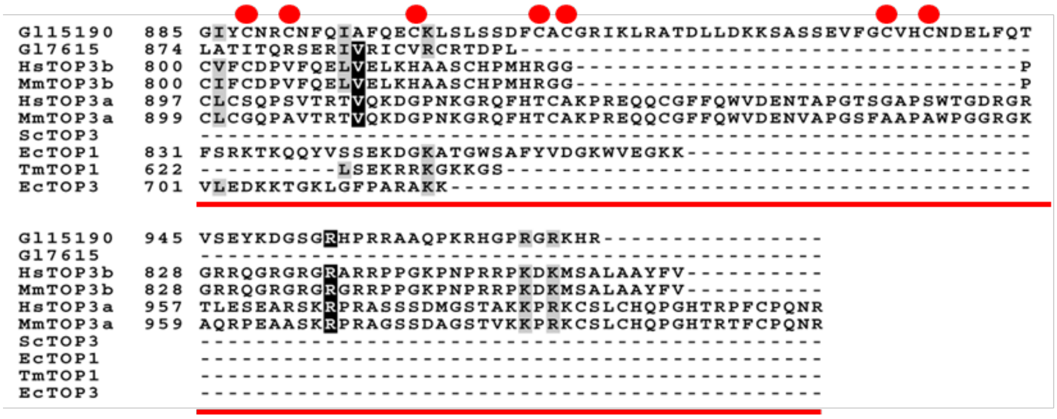

Fig. S1. Alignment of the type IA topoisomerases. The type IA topoisomerases from different orgasms, including *Mus musculus* (Mm), *Homo sapiens* (Hs), *Drosophila melanogaster* (Dm), *Saccharomyces cerevisiae* (Sc), *G. lamblia* (Gl), *Escherichia coli* (Ec), *Thermotoga Maritima* (Tm), and *Leishmania donovani* (Ld), are analyzed by ClustalW 1.83 with all default settings. GenBank *accession* numbers for MmTOP3α, MmTOP3β, HsTOP3α, HsTOP3β, DmTOP3α, DmTOP3β, ScTOP3, LdTOP3β, EcTOP1, EcTOP3, TmTOP1, Gl7615, and Gl15190 are NP_033436.1, NP_035754.1, NP_004609.1, NP_003926.1, NP_523602.2, NP_511059.2, NP_013335.1, ACX31684.1, WP_097426177.1, WP_024221956.1, WP_004082962.1, XP_001709812.1, and XM_001709742.1, respectively. Black boxes, gray boxes and hyphens indicate identical amino acids, conserved amino acids and gaps in the respective proteins, respectively. The catalytic important Tyrosine 328 is pointed by a red arrow. The decatenation loop of EcTOP3 (residues 502–519) is indicated by a purple line. The zinc ribbon domains are indicated by red lines. The cysteine residues in the zinc ribbon domains are indicated by red filled circles.

Figure S2

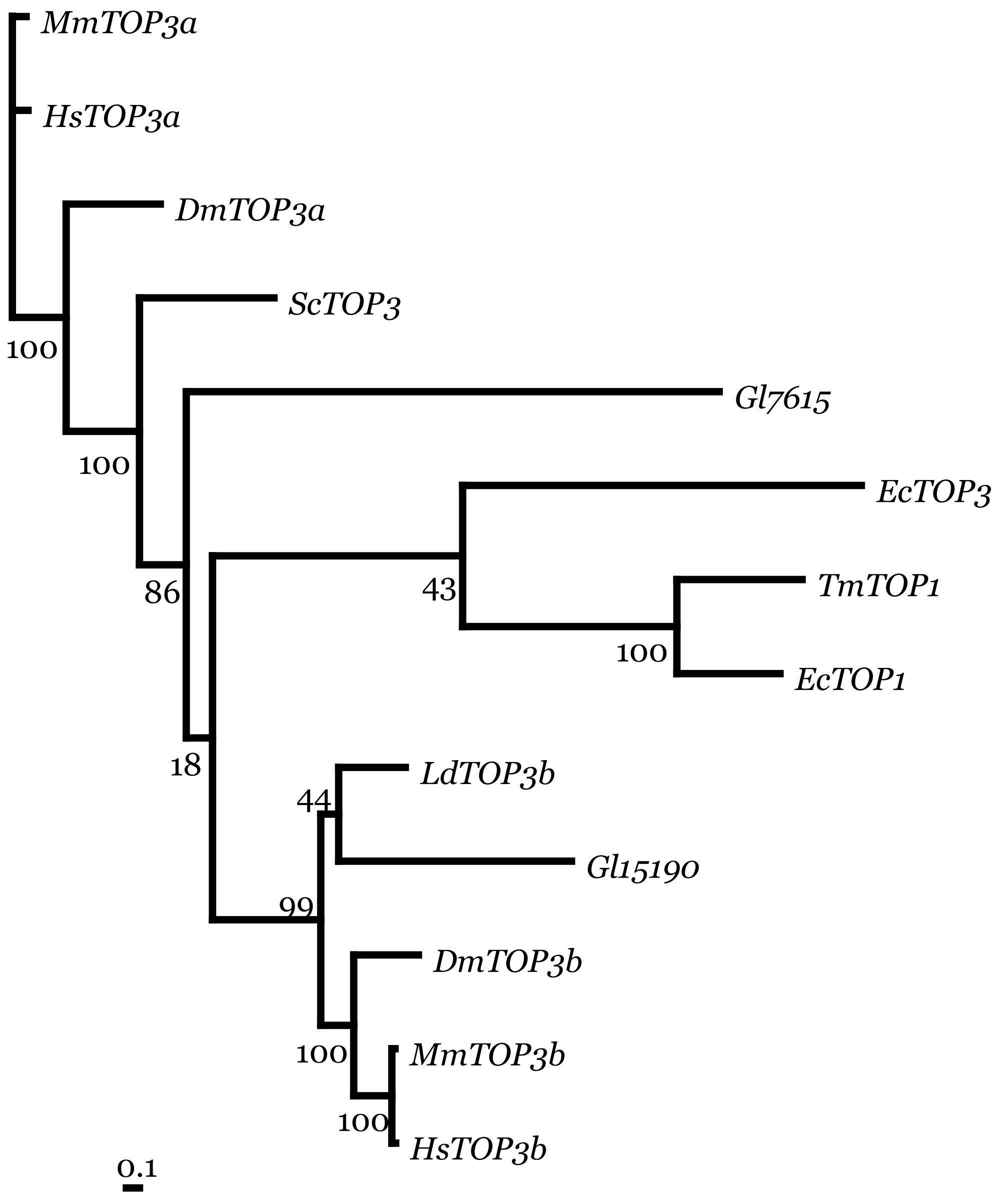

Fig. S2. Phylogenetic analysis of the type IA topoisomerases. A neighbor-joining phylogenetic tree was obtained from alignment of type IA topoisomerases from

various organisms as described. The bootstrap values determined from 1000 trees are not shown. Values are higher than 400.

Figure S3

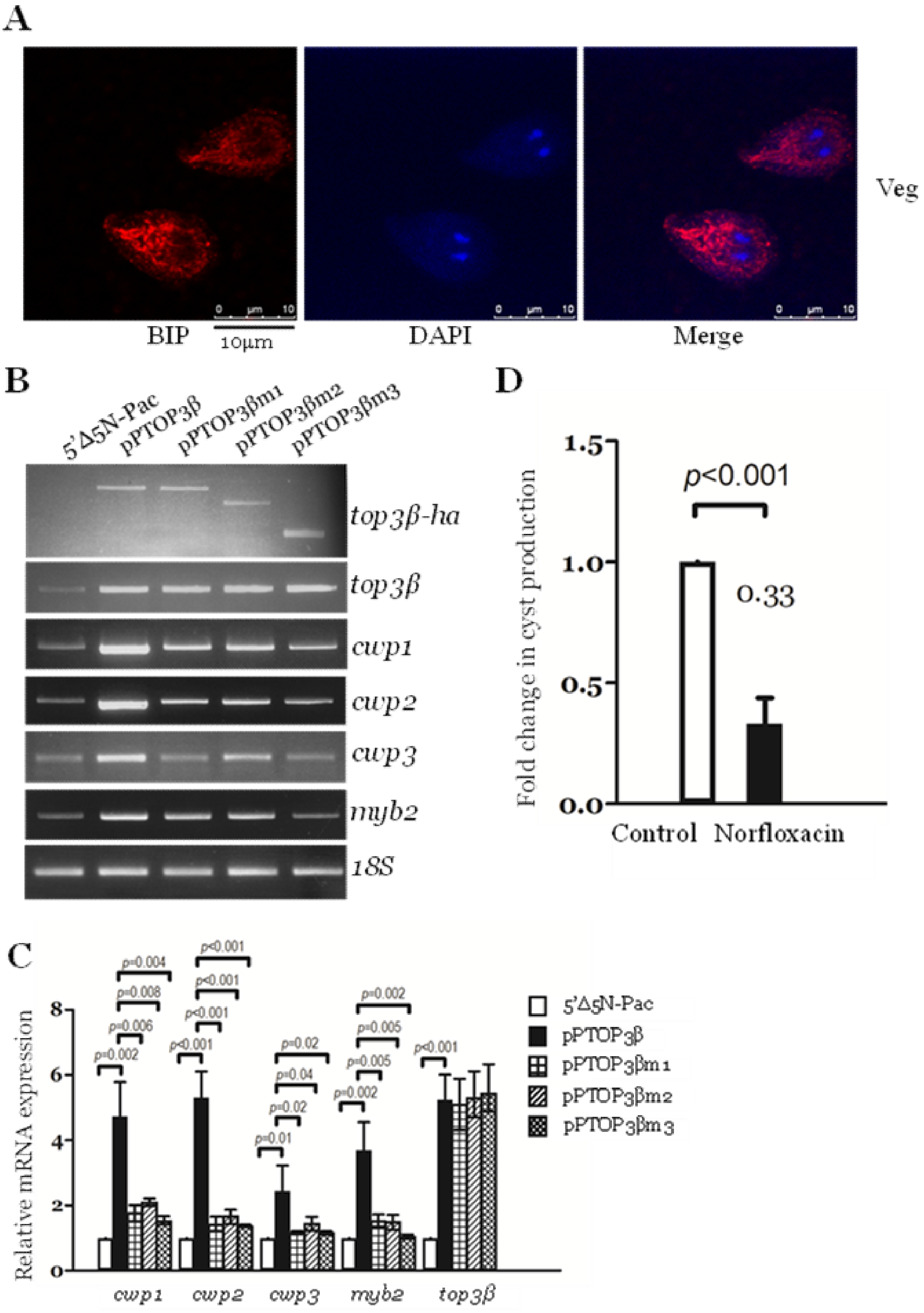
A
BIP
DAPI
Merge
Veg
0 μm 10
10μm
B
5'Δ5N-Pac
pPTOP3β
pPTOP3βm1
pPTOP3βm2
pPTOP3βm3
top3β-ha
top3β
cwp1
cwp2
cwp3
myb2
18S
C
Relative mRNA expression
0
2
4
6
8
p=0.002
p=0.006
p=0.008
p=0.004
p<0.001
p<0.001
p<0.001
p<0.001
p=0.01
p=0.02
p=0.04
p=0.02
p=0.002
p=0.005
p=0.005
p=0.002
p<0.001
cwp1
cwp2
cwp3
myb2
top3β
5'Δ5N-Pac
pPTOP3β
pPTOP3βm1
pPTOP3βm2
pPTOP3βm3
D
Fold change in cyst production
0.0
0.5
1.0
1.5
p<0.001
0.33
Control
Norfloxacin

<u>Fig. S3.</u> Induction of *cwp1-3* and *myb2* gene expression in the TOP3β-overexpressing cell line during encystation and inhibition of cyst formation by norfloxacin. (A) ER staining as determined by BIP localization. The wild-type WB trophozoites were cultured in growth medium and then subjected to immunofluorescence assays. The endogenous BIP protein was detected by anti-BIP antibody. The left panel shows that the BIP protein is localized to ER. The middle panel shows that the DAPI staining of cell nuclei. The right panel shows the merged image. The BIP staining did not overlap with DAPI. (B) RT-PCR analysis of gene expression in the TOP3β- and TOP3β mutants-expressing cell lines during encystation. The 5'Δ5N-Pac, pPTOP3β, pPTOP3βm1-3 stable transfectants were cultured in encystation medium and then subjected to RT-PCR analysis using primers specific for *top3β-ha*, *top3β*, *cwp1*, *cwp2*, *cwp3*, *myb2*, and 18S ribosomal RNA genes, respectively. Representative results are shown. (C) The intensity of bands from three RT-PCR assays was quantified using Image J. The ratio of each target gene over the loading control (18S ribosomal RNA gene) is calculated. Fold change is calculated as the ratio of the difference between the pPTOP3β/m1-3 sample and control sample, to which a value of 1 was assigned. Results are expressed as means ± 95% confidence intervals (error bars) of at least three separate experiments. $p<0.05$ was considered significant and the value was shown. (D) The addition of norfloxacin decreased cyst formation. The wild-type nontransfected WB cells were cultured in growth medium containing 497μM norfloxacin, or the same volume of Me2SO for 24h and then subjected to cyst count as described under "Materials and Methods" and Fig. 3D.

Figure S4

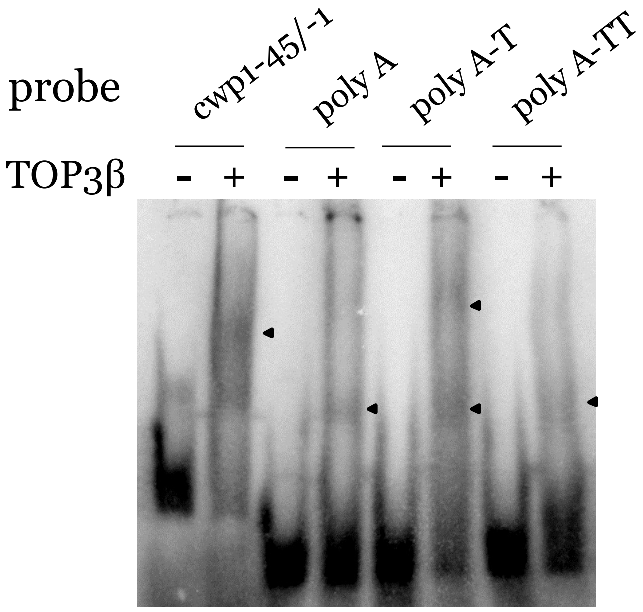

cwp1-45/-1 GTTTACAACTCTCAGATCAAGAAACACAGAAATAAAATATCAGGG
poly A AAAAAAAAAAAAAAAAAAAAAAA
poly A-T AAAAAAAAAAATAAAAAAAAAAA
poly A-TT AAAAAAAAAAATTAAAAAAAAAA

Fig. S4. TOP3β may bind to AT-rich sequence. Electrophoretic mobility shift assays were performed using purified TOP3β and various ^{32}P-end-lableled oligonucleotide probes cwp1-45/-1, poly A, poly A-T, and poly A-TT as described. Components in the binding reaction mixtures are indicated above the lanes. The arrowhead indicates the shifted complex.

Figure S5

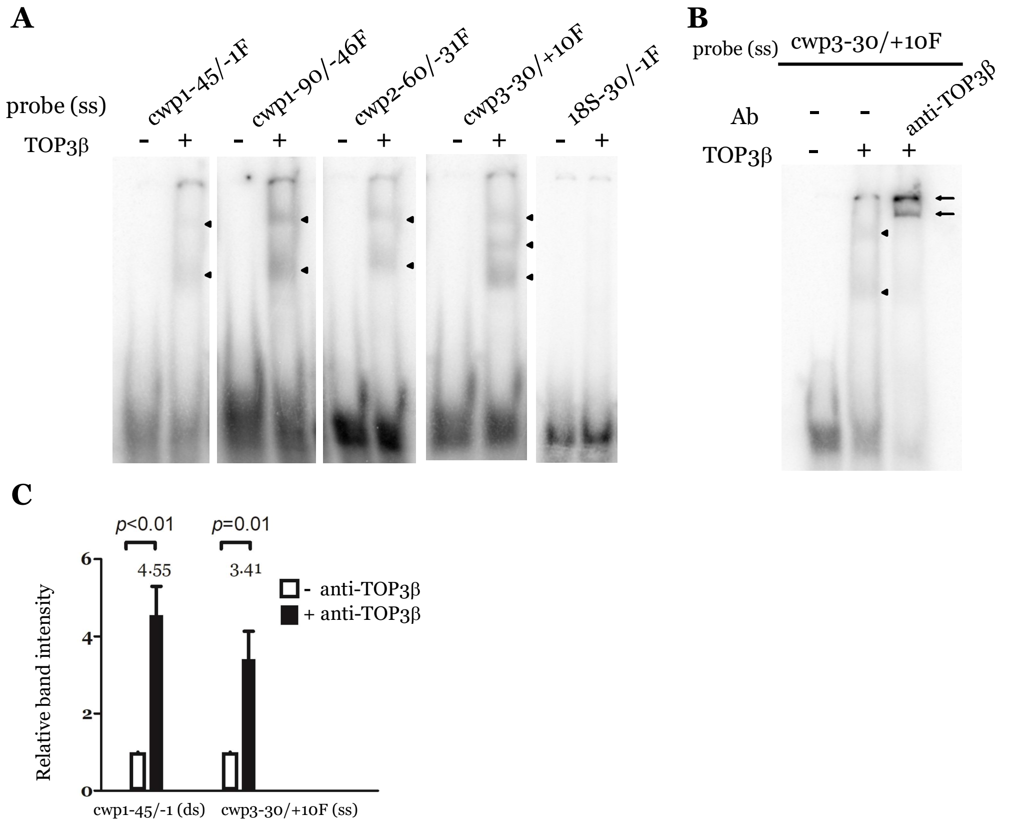

Fig. S5. Single-stranded DNA-binding and cleavage ability of TOP3β. (A) Single-stranded DNA-binding ability of TOP3β. Electrophoretic mobility shift assays were performed using purified TOP3β and ^{32}P labeled single-stranded (ss) oligonucleotide probes, such as cwp1-45/-1F (the forward single strand of the cwp1-45/-1). Components in the binding reaction mixtures are indicated above the lanes. The arrowheads indicate the shifted complexes. (B) The single-stranded DNA-binding activity of TOP3β was validated by supershift assays. Some reaction

mixtures contained 0.8μg of anti-TOP3β antibody as indicated above the lanes. (C) Quantitation of the intensity of anti-TOP3β supershift bands as indicated by arrows in Fig. 5C and Fig. S5B. The intensity of bands from three assays with double-stranded (ds) cwp1-45/-1 probe and single-stranded (ss) cwp3-40/+10F probe was quantified using Image J. Fold change is calculated as the ratio of the difference between the + anti-TOP3β sample and **-** anti-TOP3β sample, to which a value of 1 was assigned. Results are expressed as means ± 95% confidence intervals (error bars) of at least three separate experiments. $p<0.05$ was considered significant and the value was shown.

Figure S6

ATGGGCACCGAGTACAAGCCCACGGTGCGCCTCGCCACCCGCGACGACGTCCCCCGGGCCG
TACGCACCCTCGCCGCCGCGTTCGCCGACTACCCCGCCACGCGCCACACCGTCGACCCGGA
CCGCCACATCGAGCGGGTCACCGAGCTGCAAGAACTCTTCCTCACGCGCGTCGGGCTCGAC
ATCGGCAAGGTGTGGGTCGCGGACGACGGCGCCGCGGTGGCGGTCTGGACCACGCCGGAGA
GCGTCGAAGCGGGGGCGGTGTTCGCCGAGATCGGCCCGCGCATGGCCGAGTTGAGCGGTTC
CCGGCTGGCCGCGCAGCAACAGATGGAAGGCCTCCTGGCGCCGCACCGGCCCAAGGAGCCC
GCGTGGTTCCTGGCCACCGTCGGCGTCTCGCCCGACCACCAGGGCAAGGGTCTGGGCAGCG
CCGTCGTGCTCCCCGGAGTGGAGGCGGCCGAGCGCGCCGGGGTGCCCGCCTTCCTGGAGAC
CTCCGCGCCCCGCAACCTCCCCTTCTACGAGCGGCTCGGCTTCACCGTCACCGCCGACGTC
GAGTGCCCG**AAGGACCGCGCGACCTGG**TGCATGACCCGCAAGCCCGGTGCCCTCGAGTGAc
catcaagtgcttgctattatgtaaactacttcctactccattaaatatttagacgcgcagt
tctggctatgggaccgaaacttttgaagagcgagtcagctatgagtttatggatgaaaacg
cagttcaatcagatcgtgcggtatataagtataagatacataaccgtagagtgctaatcac
gacccaccgagtagttgataattcatcaatcactggcagggcaaatcactcaccagaacca
gctagttcaatttgtgcttcggactttagatctgctgatctagactttattccactaacgg
atacttttagatcagttgttcaccacttggtggtactctctgtgacgtcaccttgtgcgtg
atgcagtgccccagttccgcctgttgctgctgccaatggaggcccgccataacgttctacg
cagtctttctgatcagccttgcgctccaactggcgacagaggagtgcacaaccgtctctgg
tgatcagcccgacacttgcaaggcctgtagcgccgtcatcaacggcaagaagtactgctcc
cagtgcaacggcggtgggtctcaatctgccccaacggacggagtgtgctcagcagacaaca
acgaatgcccctaaaagaacaacggagtatgtacacagtgcgctcatgagtccttcatgta
caagagcggatgctacaaggatagccaggcacctggcaacacgatgtgtgaaacagcaact
gatggagtgtgcacactaactaaggctggatacttcgtgccgccgggcgcagacgcctctc
accagt**cggtcataccatgtggagacg**

Fig. S6. Replacement of the *top3β* gene with the *pac* gene in the TOP3βtd cell line confirmed by PCR and sequencing. Genomic DNA was isolated from the TOP3βtd and control cell lines cultured in growth medium. PCR was performed using primers specific for *pac* (PCR2 in Fig. 8A), which are PCR2F for bold region 1 and PCR2R for bold region 2, to verify the integration of the *pac* gene into the correct region in genomic DNA. The sequence results obtained from the PCR2 product are shown as underlined letters. Capital letters indicate the coding sequence for the *pac* gene, which

starts at ATG and stops at TGA. This indicates the replacement of the *top3β* gene with the *pac* gene. The region used to clone the *top3β* 3' region into the pTOP3βtd plasmid for HR is shown in red, which is also between the sequence of top3β3XF and top3β3KR. The underlined and lower case letters, which are downstream and outside of the red region of top3β3XF and top3β3KR, indicate that HR occurred in the sequence of *top3β* 3' region and that the *pac* gene was integrated in the genomic DNA.

Figure S7

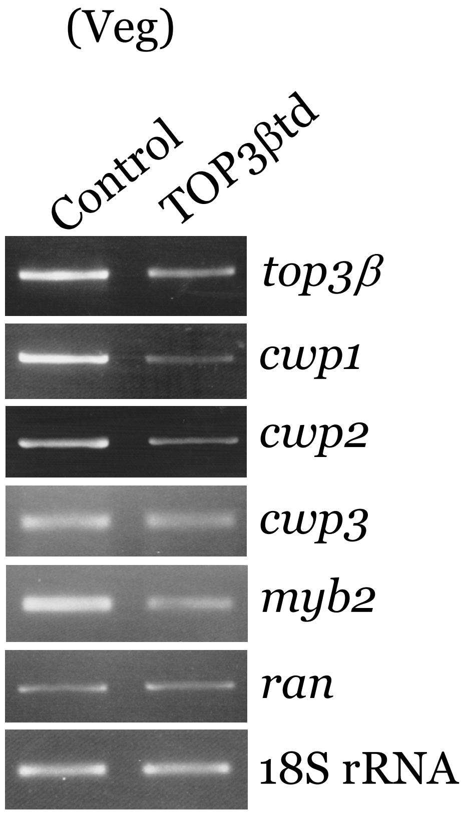

Fig. S7. RT-PCR analysis of gene expression in the TOP3βtd cell line. The control and TOP3βtd cell lines were cultured in growth medium and then subjected to RT-PCR analysis using primers specific for *top3β*, *cwp1*, *cwp2*, *cwp3*, *myb2*, *ran*, and 18S ribosomal RNA genes, respectively. The *ran* mRNA levels did not significantly change.

Figure S8

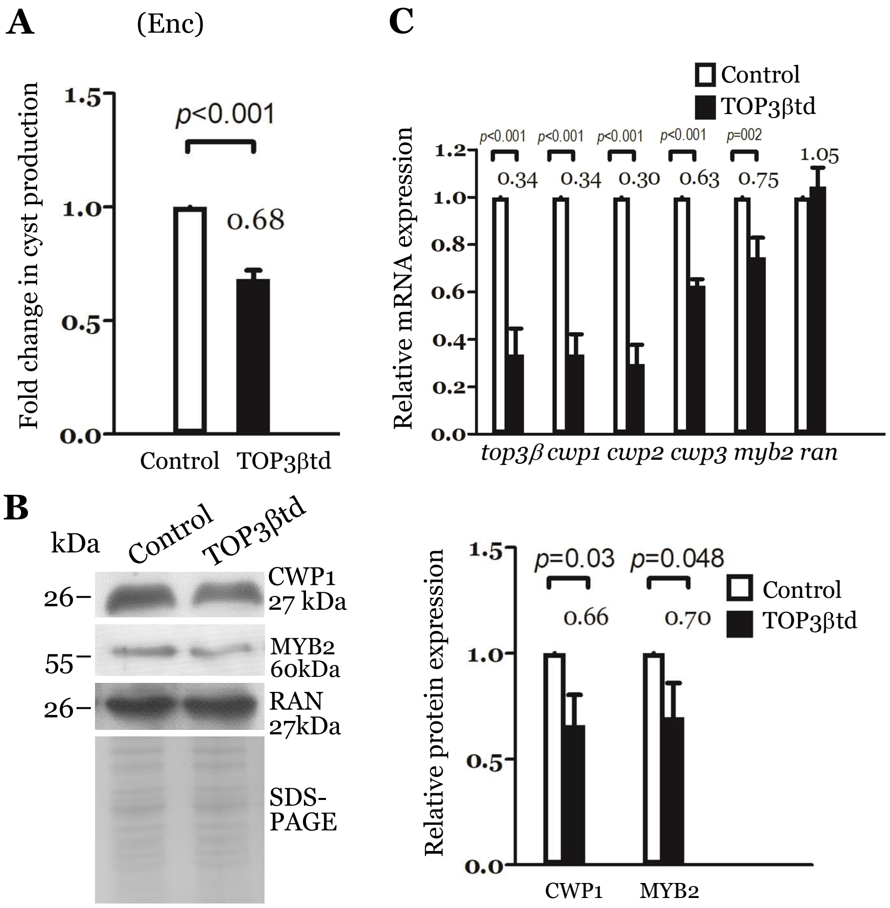

Fig. S8. Decrease in expression of *cwp1-3* and *myb2* by targeted disruption of the *top3β* gene during encystation. (A) Cyst formation decreased by targeted disruption of the *top3β* gene in the TOP3βtd cell line during encystation. The control and TOP3βtd cell lines were cultured in encystation medium for 24h (Enc) and then subjected to cyst count as described under "Materials and Methods" and Fig. 3D. (B) Targeted disruption of the *top3β* gene decreased the CWP1 and MYB2 levels in the TOP3βtd

cell line during encystation. The control and TOP3βtd cell lines were cultured in encystation medium for 24h and then subjected to SDS-PAGE and Western blot analysis as described in Fig. 3A. The blot was probed with anti-CWP1, anti-MYB2, and anti-RAN antibodies, respectively. The intensity of bands from three Western blot assays was quantified as described in Fig. 3A. (C) Decrease in expression of *cwp1-3* and *myb2* by targeted disruption of the *top3β* gene in the TOP3βtd cell line during encystation. The control and TOP3βtd cell lines were cultured in encystation medium for 24h and then subjected to quantitative real-time RT-PCR analysis using primers specific for *top3β*, *cwp1*, *cwp2*, *cwp3*, *myb2*, *ran*, and 18S ribosomal RNA genes, respectively, as described in Fig. 1B.

Figure S9

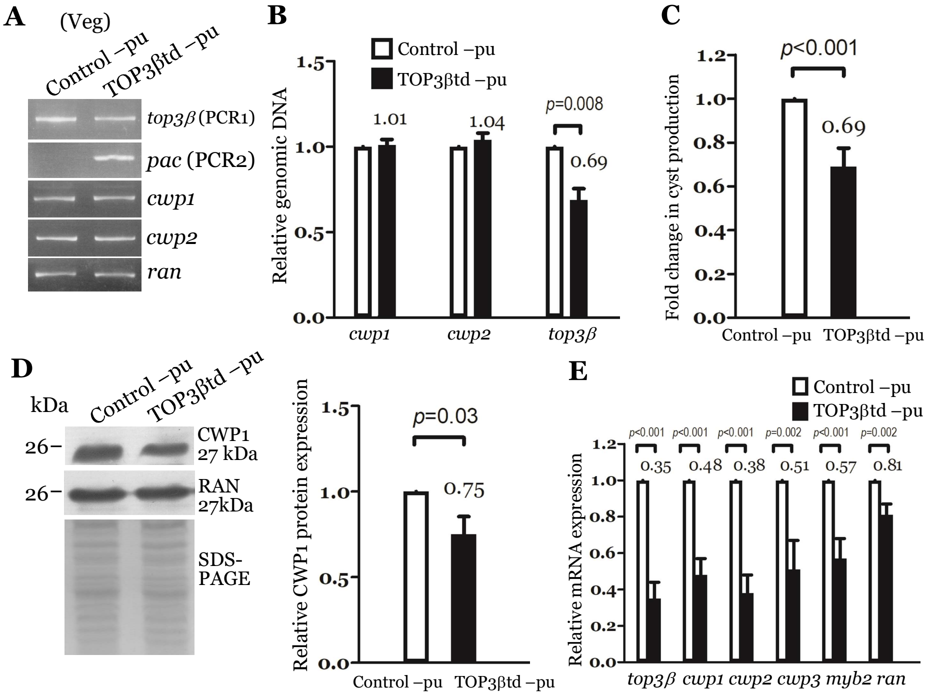

<u>Fig. S9.</u> Decrease in expression of *cwp1-3* and *myb2* by targeted disruption of the *top3β* gene after the removal of puromycin during vegetative growth. (A) Partial replacement of the *top3β* gene with the *pac* gene in the TOP3β –pu cell line confirmed by PCR. Puromycin was removed from the TOP3βtd and control cell lines to obtain the TOP3βtd –pu and control –pu cell lines, respectively. Genomic DNA was isolated from the TOP3βtd –pu and control -pu cell lines cultured in growth medium (vegetative growth, Veg). PCR was performed using primers specific for *top3β* (PCR1), *pac* (PCR2), *cwp1*, *cwp2*, and *ran* genes, respectively, as described in Fig. 8B. (B) Partial disruption of the *top3β* gene in the TOP3βtd -pu cell line confirmed by real-time PCR. Real-time PCR was performed using genomic DNA and primers specific for *top3β*, *cwp1*, *cwp2*, and *ran* genes, respectively, as described in

Fig. 8C. (C) Cyst formation decreased by targeted disruption of the *top3β* gene in the TOP3βtd –pu cell line during vegetative growth. The control –pu and TOP3βtd –pu cell lines were cultured in growth medium and then subjected to cyst count as described under "Materials and Methods" and Fig. 3D. (D) Targeted disruption of the *top3β* gene decreased the CWP1 and MYB2 levels in the TOP3βtd –pu cell line during vegetative growth. The control –pu and TOP3βtd –pu cell lines were cultured in growth medium and then subjected to SDS-PAGE and Western blot analysis as described in Fig. 3A. The blot was probed with anti-CWP1, anti-MYB2, and anti-RAN antibodies, respectively. The intensity of bands from three Western blot assays was quantified as described in Fig. 3A. (E) Decrease in expression of *cwp1-3* and *myb2* by targeted disruption of the *top3β* gene in the TOP3βtd –pu cell line during vegetative growth. The control –pu and TOP3βtd –pu cell lines were cultured in growth medium and then subjected to quantitative real-time RT-PCR analysis using primers specific for *top3β*, *cwp1*, *cwp2*, *cwp3*, *myb2*, *ran*, and 18S ribosomal RNA genes, respectively, as described in Fig. 1B.

Figure S10

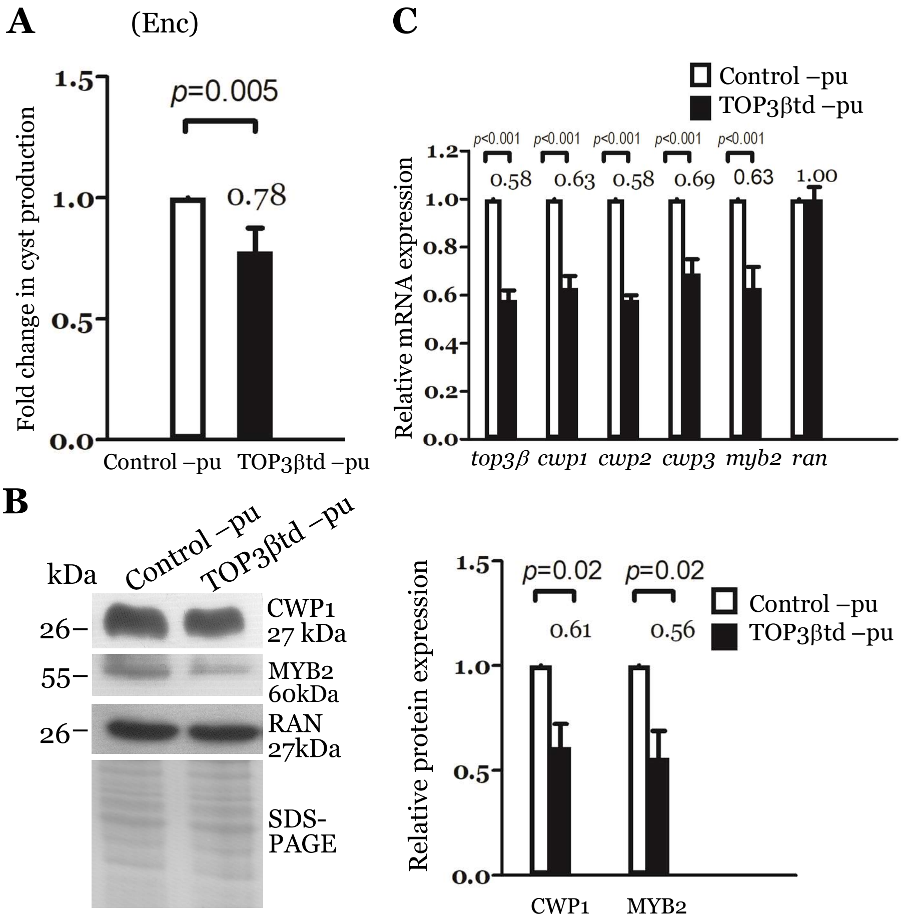

D

-200
TCCTGCACGCTTTGAGAAGGAATTGGCGGCCGGATATGTGTCCCCCAAACGGGCTTCACTCGGG

TGCGCCAGCGAACGAGTTTGGGGGATGATGGTATCTTCCCCTGTAGCTTTCTTAAAGGGCTACAC

CATCTCGCATAGTTGTCACTCTTACTACTTTTCTTCCTATGGGGCTGAGGGCTGGGATGC**TAAA**

-1
AATAATTC

Fig. S10. Decrease in expression of *cwp1-3* and *myb2* by targeted disruption of the *top3β* gene after the removal of puromycin during encystation. (A) Cyst formation decreased by targeted disruption of the *top3β* gene in the TOP3βtd –pu cell line during encystation. The control –pu and TOP3βtd –pu cell lines were cultured in

encystation medium for 24h (Enc) and then subjected to cyst count as described under "Materials and Methods", as described in Fig. 3D. (B) Targeted disruption of the *top3β* gene decreased the CWP1 and MYB2 levels in the TOP3βtd –pu cell line during encystation. The control –pu and TOP3βtd –pu cell lines were cultured in encystation medium and then subjected to SDS-PAGE and Western blot analysis as described in Fig. 3A. The blot was probed with anti-CWP1, anti-MYB2, and anti-RAN antibodies, respectively. The intensity of bands from three Western blot assays was quantified as described in Fig. 3A. (C) Decrease in expression of *cwp1-3* and *myb2* by targeted disruption of the *top3β* gene in the TOP3βtd –pu cell line during encystation. The control –pu and TOP3βtd –pu cell lines were cultured in encystation medium and then subjected to quantitative real-time RT-PCR analysis using primers specific for *top3β*, *cwp1*, *cwp2*, *cwp3*, *myb2*, *ran*, and 18S ribosomal RNA genes, respectively, as described in Fig. 1B. (D) The MYB2 binding site in the *top3β* promoter. The 200-bp 5' untranslated region of the *top3β* promoter is shown. The AT-rich promoter element is in bold. The underlined sequence, CTGTAG, is the reverse sequence of the MYB2 binding sequence, CTACAG.
